# Endogenous Fms-like Tyrosine Kinase-3 Ligand levels are not altered in mice after a severe burn and infection

**DOI:** 10.1186/1471-2172-10-47

**Published:** 2009-08-28

**Authors:** Julia K Bohannon, Weihua Cui, Tracy Toliver-Kinsky

**Affiliations:** 1 Department of Microbiology and Immunology, University of Texas Medical Branch, Galveston, TX, USA; 2 Department of Anesthesiology, University of Texas Medical Branch, Galveston, TX., USA

## Abstract

**Background:**

Fms-like tyrosine kinase-3 ligand (Flt3L) is a hemopoietic cytokine and dendritic cell (DC) growth factor that promotes the proliferation and differentiation of progenitor cells into DCs. We have previously found that treatment of severely burned mice with recombinant Flt3L significantly enhances DC production and bacterial clearance from infected burn wounds, and increases global immune cell activation and survival in response to a burn wound infection. These significant benefits of Flt3L treatment after burn injury have prompted the question of whether or not severe burn injury induces deficits in endogenous Flt3L levels that could affect DCs and subsequent responses to infection.

**Results:**

To address this, male BALB/c mice received a 30% total body surface area scald burn. Blood, spleens, and wound-draining lymph nodes were harvested at various time-points after injury. Some mice received a wound inoculation with *P. aeruginosa*. Murine Flt3L and G-CSF levels were measured by ELISA. Burn injury had no significant effect on Flt3L levels at any post-burn time-point examined compared to normal Flt3L levels in the sera, spleen, or lymph nodes. Additionally, Flt3L levels in the sera, spleen, and lymph nodes were not significantly altered when wounds were inoculated on the day of burn injury or at post-burn time points examined. Alternatively, levels of G-CSF were increased in response to burn injury and burn wound infection. Additionally, DC numbers and functions were not altered following burn injury alone. There was no significant difference between the number of DCs in the spleens of sham-injured mice and mice at 5 days after burn injury. When naïve T cells from sham-injured mice were co-cultured with DCs from either sham- or burn-injured mice, IFN-γ production was similar, however, IFN-γ levels produced by T cells harvested from burn-injured mice were significantly lower than those produced by T cells from sham mice, regardless of which DC group, sham or burn, was used in the coculture.

**Conclusion:**

These data suggest that the beneficial effects of Flt3L treatments after burn injury are not due to correction of a burn-associated Flt3L deficiency but rather, are likely due to supplementary stimulation of DC production and immune responses to infection.

## Background

Severe burn injury is known to perturb hematopoietic and immune cell homeostasis. These perturbations can decrease the efficacy of immune responses to infection, which is a frequent problem for burned patients. Innate immune responses following severe burn injury are associated with impairments in the functions of natural killer cells, neutrophils, and antigen presenting cells, all of which are crucial for the establishment of a normal response to infection [1-4]. The production of various innate immune cells from their hematopoietic precursors is also impacted by burn injury. It has been reported that burn injury with sepsis causes a reduction in the proliferative capacity of bone marrow progenitor cells that give rise to granulocytes, and a relative increase in monocytopoiesis [5,6]. Others have reported that burn injury alone increases numbers of monocyte progenitor cells and the production of inflammatory monocytes and also increases granulocyte progenitors and neutrophils in the spleen [7,8]. Transient decreases in bone marrow neutrophil numbers, subsequent to neutrophil egress into the circulation after burn injury, have also been reported [9]. Muthu *et al*. demonstrated an impairment, induced by both burn injury alone and burn with sepsis, in the *in vitro *differentiation of myeloid dendritic cells (DCs) from monocyte progenitor cells [10].

Hematopoietic cytokines that regulate leukocyte generation can also be altered by burn injury. Granulocyte-colony stimulating factor (G-CSF), which promotes neutrophil production, has been shown to be elevated following severe burns in both murine models of injury and in human burn patients. Similarly, granulocyte macrophage-colony stimulating factor (GM-CSF), a cytokine that promotes production and activation of neutrophils and macrophages, is also elevated in burn patients and murine models [7,11-13]. Fms-like tyrosine kinase-3 ligand (Flt3L) is a hematopoietic cytokine that promotes the production of DCs from both myeloid and lymphoid lineage-committed progenitor cells. We have previously reported that human Flt3L can be used to enhance DC production in burn-injured mice and increase resistance to infections [14,15]. While treatments with exogenous Flt3L are protective, the effects of burn injury on endogenous Flt3L levels are not known. This study was conducted to determine the effects of burn injury alone, or following wound infection, on endogenous levels of murine Flt3L.

## Results

### Endogenous Flt3L and G-CSF levels after burn injury

**Figure 1:**
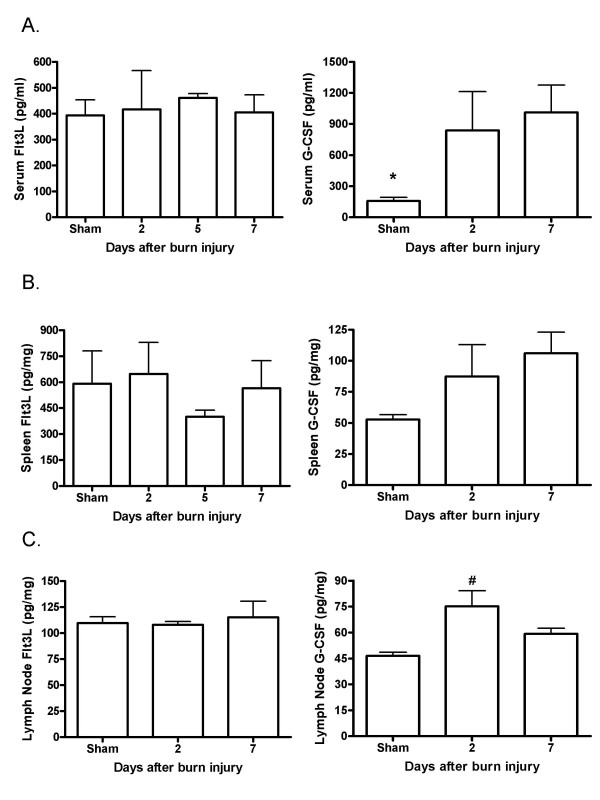
**Endogenous Flt3L and G-CSF levels after burn injury**. Sera, spleens, and lymph nodes were harvested at various times post-burn, or from sham-injured mice. Endogenous Flt3L and G-CSF levels were measured by ELISA. Data shown are the means +/- SD. *significantly different from all others; #significantly different from sham, p < 0.05, n = 3 - 6 mice/group.

Flt3L was measured in the serum, spleen, and wound-draining lymph node homogenates at various times following a severe burn injury. There were no significant changes in serum or wound-draining lymph node levels of Flt3L in response to the injury alone at any of the time points examined (figure 1). Although there appeared to be a trend towards decreased Flt3L in the spleen at 5 days post-burn, no significant differences between splenic Flt3L levels were noted for any of the time points examined. As the levels of G-CSF and GM-CSF have been shown to be either unaffected or elevated following severe burn injury, we also examined these cytokines to confirm consistency of our model with others reported in the literature [7,11-13]. We found that levels of GM-CSF were not significantly altered by burn injury (data not shown). However, levels of G-CSF were significantly elevated following burn injury alone (figure 1). Specifically, serum levels of G-CSF were significantly increased 2 days after a burn injury and remained significantly elevated at 7 days post-burn. Although there was a trend towards increased levels of G-CSF in the spleen following burn injury, the differences were not significant. G-CSF levels in the draining lymph nodes were also significantly elevated at 2 days post-burn.

### Endogenous Flt3L and G-CSF levels after burn wound infection

**Figure 2:**
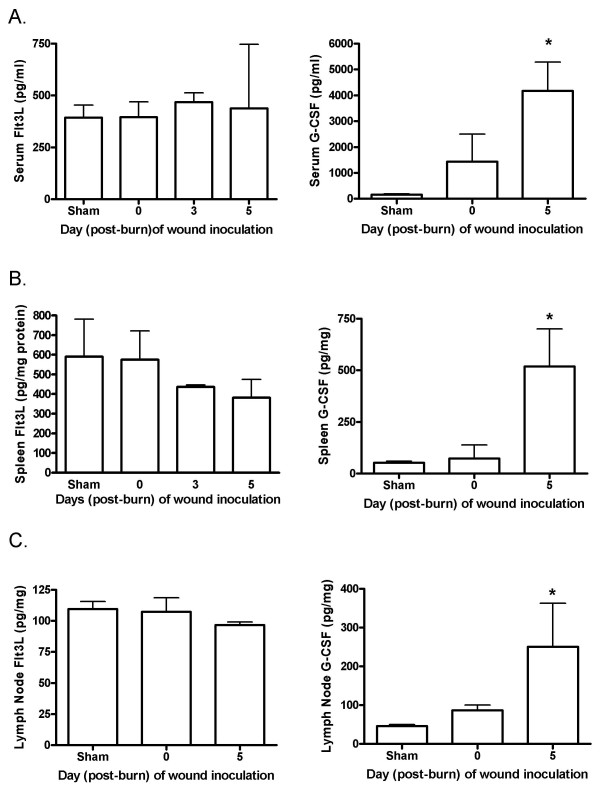
**Endogenous Flt3L and G-CSF levels after a burn wound infection**. Sera, spleens, and lymph nodes were harvested at various times post-burn, two days following inoculation of burn wounds. Endogenous Flt3L and G-CSF levels were measured by ELISA. Data show means +/- SD. *significantly different from all others, p < 0.05, n = 3 - 6 mice/group.

To determine if a wound infection induces changes in systemic Flt3L levels after a burn injury, wounds were inoculated with *P. aeruginosa *two days before tissue harvest at various time points post-burn. In this model, mortalities begin to occur 3 days following wound inoculation due to widespread systemic dissemination of bacteria. Therefore, Flt3L was measured 2 days following wound inoculation, before morbidity occurs, and the inocula used at each time point produced similar responses and levels of mortality [14,15]. There were no significant changes in Flt3L levels in sera or wound-draining lymph nodes in response to a burn wound infection regardless of the time point of infection or harvest (figure 2). While there appeared to be a decrease in splenic Flt3L levels in response to a wound inoculation at 3 and 5 days post-burn, the differences were not significant. However, G-CSF levels were significantly elevated in all tissues examined in response to a wound inoculation at 5 days post-burn (figure 2). GM-CSF levels were not altered in response to infection (data not shown).

### DC numbers and functions are not impaired after burn injury

**Figure 3:**
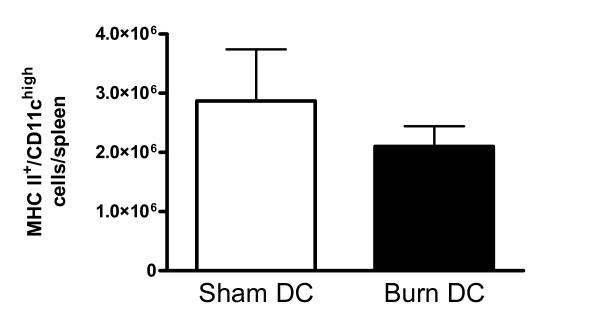
**Splenic dendritic cell numbers are not altered by burn injury alone**. Spleens were harvested 5 days after sham or burn injuries. Cell suspensions were stained with antibodies and analyzed by flow cytometry. MHC-II^+^/CD11c^high ^cells were considered to be DCs. Data show means +/- SD, n = 3 mice/group.

Others have demonstrated that sepsis can induce loss of DCs in the spleen and lymph nodes, but the effects of burn injury alone on DCs are less known [16,17]. Figure 3 shows that there was no significant difference between the number of DCs in the spleens of sham-injured mice and mice at 5 days after burn injury. Additionally, burn injury did not alter the proportions of specific DC subtypes, including plasmacytoid DCs, CD8^+ ^DCs, or CD11b^+ ^DCs (data not shown).

**Figure 4:**
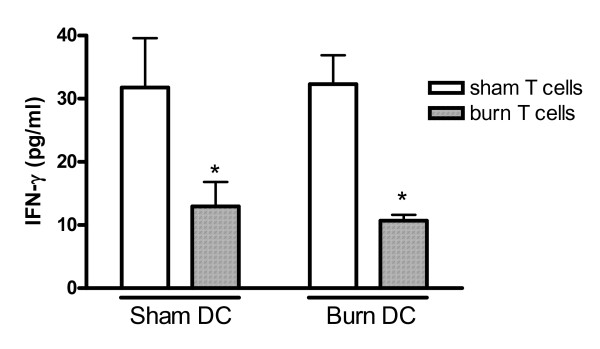
**Impaired IFN-γ production in DC-T cell cocultures is due to altered T cell, but not DC, function after burn injury**. Cells were prepared from mouse spleens 5 days after sham or burn injuries. Naïve T cells, co-cultured with HKPA-stimulated DCs, were stimulated with Concanavalin A and media were harvested 24 hours later. Cells were isolated from 3 mice and cultures were performed in quadruplicate. IFN-γ levels were measured in co-culture supernatants by ELISA. Data show means +/- SD. *significantly different from cultures containing sham T cells, p < 0.05.

We previously reported that burn-associated impairment of IFN-γ production *in vitro *is predominantly associated with impairments in lymphocytes [18]. These earlier studies were performed using mixed adherent cells, of which DCs constitute a relatively low proportion. Therefore, to determine if burn injury affects the ability of DCs to promote IFN-γ responses, we examined IFN-γ production in co-cultures using purified DCs. When naïve T cells from sham-injured mice were co-cultured with DCs from either sham- or burn-injured mice, IFN-γ production was similar (figure 4). However, IFN-γ levels produced by T cells harvested from burn-injured mice were significantly lower than those produced by T cells from sham mice, regardless or which DC group, sham or burn, was used in the co-culture. IFN-γ levels were below detection limits of the assay in cultures containing DCs or T cells alone (data not shown).

## Discussion

This study demonstrates that levels of endogenous Flt3L in the circulation, the spleen, and in wound draining lymph nodes are not significantly altered within a week following a severe burn injury. Similarly, Flt3L levels were not significantly altered when the burn injury was complicated by a wound infection. This is in contrast to other hematopoietic cytokines such as G-CSF and GM-CSF, both of which are significantly elevated in the circulation after burns [7,11-13]. Cytokine responses to burn injury are most pronounced during the first week, when injured tissues produce inflammatory mediators that can activate systemic inflammation. G-CSF and GM-CSF have been associated with regulation of inflammatory responses whereas there is little known about regulation of endogenous Flt3L production. It has been reported that translocation of Flt3L from intracellular stores to the surface of T cells occurs following hematopoietic failure induced by chemoradiation but there are no reports of Flt3L responses during inflammation [19]. Since severe burns, and especially wound infections, are known to induce systemic inflammatory responses, the data presented here suggest that endogenous Flt3L production is not associated with inflammatory responses but is mostly involved in maintenance of hematopoietic homeostasis.

Consistent with steady levels of Flt3L following severe burn injury, there were no significant changes noted in the numbers of splenic DCs after burn injury, nor in the proportions of various DC subtypes. It has already been noted that severe sepsis induces depletion of DCs, but this response was not observed following burn injury alone, or during the early responses to a burn wound infection. Muthu *et al*. demonstrated an impairment, induced by both burn injury alone and burn with sepsis, in the capacity of monocyte progenitor cells to differentiate into myeloid dendritic cells *in vitro *[10]. However, DCs are produced *in vivo *from multiple precursor pools, and production from each is differentially regulated and produces phenotypically distinct populations. In addition to differentiating from monocytes in response to the cytokines GM-CSF and IL-4, DCs also arise from progenitor cells in other tissues. In response to GM-CSF and TNF-α, myeloid DCs can be induced from cells in the bone marrow, cord blood, and peripheral blood [20]. DCs can also arise from lymphoid tissues, including the thymus, spleen, and lymph nodes in response to similar cytokine signals. In this study, we saw no effects of burn injury on the relative proportions of CD8^+ ^(lymphoid-related), CD11b^+ ^(myeloid-related), or plasmacytoid DC subsets. Other studies have shown sepsis-induced depletion of DCs in lymphoid tissues, including the lymph nodes and spleen [16,17]. We do not see any alteration in DC numbers or function during the early stages of wound infection but it is likely that depletion of DCs would follow the onset of severe sepsis. Similarly, it is possible that endogenous Flt3L levels would be altered by severe sepsis, but only early responses to wound infection preceding sepsis were examined.

Similarly, we saw no effect of burn on the ability of DCs to promote Th1-type cytokine production. DCs from burned mice were able to induce IFN-γ production by T cells similarly to DCs from sham mice. While IFN-γ production was impaired after burn injury, our results demonstrate that this impairment is not due to altered DCs but rather to T cells. It has been proposed that enhanced IL-10 production contributes to suppressed IFN-γ production after burn injury, as neutralization of IL-10 can restore IFN-γ levels, while others have suggested that suppressed Th1 responses are due not to increased IL-10, but rather to a global T cell anergy resulting from insufficient activation by DCs secondary to decreased differentiation of DCs from monocyte precursors after burn injury [18,21,22]. We did not investigate either of these potential causes as a source of impaired IFN-γ, since burn-associated impairments were only detected in co-cultures containing T cells from burned mice, but not DCs, which were the focus of the study. While alterations in DC promotion of IFN-γ production were not noted in this study, potential effects of burn injury on other DC functions cannot be excluded.

Similar to Flt3L, Gamelli and colleagues reported that the levels of endogenous G-CSF are also not impaired by burn wound infection [7,11-13]. In fact, G-CSF levels have shown to be significantly increased in the bone marrow, spleen, and serum of burn and burn-infected animals. Consistent with these findings, we demonstrate that G-CSF levels are significantly elevated in the wound-draining lymph nodes, the spleen, and the sera of burned and burned infected mice in our model. Interestingly, contraindicative to this finding, burn and infection lead to enhanced neutropenia. It has been suggested that the presence of increased G-CSF values and numbers of granulocyte-macrophage colony-forming cells (GM-CFCs), along with the simultaneous presence of neutropenia in burn and burn-infected animals is due to other immune alterations resulting from burn injury, including reduced G-CFC responsiveness to G-CSF, altered cell-cycle of GM-CFCs by increased levels of PGE_2_, and a shift from granulocytopoiesis to monocytopoiesis, rather than an impairment in endogenous G-CSF levels. It is thought that the benefits of exogenous G-CSF treatment after burn injury is due to a reversal of these impaired immune responses [13]. Similarly, the finding that endogenous Flt3L levels are not changed with burn or infection suggests that the previously reported protective effects provided by exogenous Flt3L treatments after burn injury are mediated through enhancement of impaired immune functions, rather than a restoration of impaired Flt3L levels.

## Conclusion

Treatment with exogenous Flt3L after burn injury increases survival in a mouse model of lethal burn wound infection. This study sought to determine if endogenous Flt3L levels are altered by burn injury and burn wound infection. The results show that neither burn injury nor post-burn wound infection alter endogenous Flt3L levels. Additionally, DC numbers and function are not impaired by burn injury alone. Therefore, it appears that the benefits of Flt3L treatment following burn injury are not due to a restoration of burn-induced endogenous Flt3L deficiency. Rather, Flt3L treatments following burn injury likely provide protection against mortality due to infection through supplementary enhancement of DC production and differentiation, along with enhancement of the immune response to infection.

## Methods

### Burn Model

All animal procedures were consistent with the National Institutes of Health guidelines for the care and use of experimental animals and were approved by the Institutional Animal Care and Use Committee at the University of Texas Medical Branch. A full-thickness scald burn was used as previously described [15]. Briefly, male BALB/c mice, 6-8 weeks of age, were anesthetized with 2.5% isoflurane, and shaved on the dorsal and lateral surfaces. Mice were placed on their backs and secured in a protective template with an opening corresponding to 35% of the total body surface area, and the exposed skin was immersed for 10 seconds in 97°C water. Lactated Ringers solution (2 ml) was injected i.p. and buprenorphine (2 mg/kg) was given for analgesia. Sham-injured mice were handled identically except for immersion in water.

### Infection

*P. aeruginosa*, a common source of infections in burn patients, was purchased from American Type Culture Collection (ATCC#19660) [23-25]. Cultures were grown in tryptic soy broth and diluted in sterile saline solution for wound inoculation. Two days prior to sacrifice, a lethal dose of *P. aeruginosa *was applied to the surface of the wound. Blood, spleens, and wound-draining lymph nodes were harvested at various times after injury. Mice harvested at 2 days post-burn were inoculated with 500 cfu on the day of injury; those harvested at 5 days post-burn were inoculated with 8000 cfu 3 days after injury; and mice harvested at 7 days post-burn were inoculated with 10^4 ^cfu 5 days after injury. Each of these doses induces a comparable level of mortality when applied to the wound at the respective days after injury [14,15]. This design permitted examination of responses to burn injury, with and without infection, at similar time points and kept the lethality of infection similar between groups.

### Endogenous Flt3L Measurements

Flt3L levels were measured in sera and spleen and lymph node homogenates using the Quantikine Mouse Flt-3 Ligand Immunoassay by R&D Systems according to manufacturer recommendations. Spleens and lymph nodes were prepared for ELISA by homogenization in RIPA buffer (25 mM Tris-HCl, pH 7.6, 150 mM NaCl, 1% NP-40, 1% sodium deoxycholate, 0.1% SDS) supplemented with complete protease inhibitor cocktail (Roche Applied Science, Indianapolis, IN), followed by centrifugation and measurement of proteins in the supernatant. Proteins were measured using the Bio-Rad protein dye reagent (Bio-Rad, Hercules, CA).

### G-CSF Measurements

G-CSF levels were measured in the sera and spleen and lymph node homogenates using the Mouse G-CSF ELISA Kit by R&D Systems according to manufacturer recommendations. Skin, spleen, and lymph nodes were prepared for ELISA by homogenization in RIPA buffer (25 mM Tris-HCL, ph 7.6, 150 mM NaCl, 1% sodium deoxycholate, 0.1% SDS) supplemented with complete protease inhibitor cocktail (Roche Applied Science, Indianapolis, IN).

### Dendritic Cell Measurements

Spleens were harvested 5 days after sham or burn injuries and single cell suspensions were prepared as previously described [18,26]. To determine DC numbers, cell suspensions were incubated at 4°C for 20 minutes with fluorescently-conjugated antibodies specific for class II MHC and CD11c, CD8, CD11b, and mPDCA-1, washed in PBS, fixed in 1% buffered (ph 7.8) paraformaldehyde, then analyzed on a FACSort flow cytometer (Becton Dickenson; Franklin Lakes, NJ). Specific staining was determined by comparison with appropriate antibody isotype controls. Antibodies were purchased from BD Biosciences (San Jose, CA). MHC-II^+^/CD11c^high ^cells were considered to be DCs.

For DC-T cell cocultures, CD11c^+ ^DCs were positively selected using magnetic beads and separation columns (Miltenyi Biotec; Auburn, CA). Naïve T cells (CD4^+^/CD62L^+^/CD44^low^) were isolated using enrichment columns from R&D Systems (Minneapolis, MN). DCs were activated *in vitro *overnight with heat-killed *P. aeruginosa*, washed, then co-cultured with T cells (10^4 ^dendritic cells:10^5 ^T cells) for 5 days. Cultures were stimulated with Concanavalin A (1 mg/ml) and media were harvested 24 hours later for measurement of IFN-γ levels by ELISA (eBioscience, San Diego, CA).

### Statistics

All statistical analyses were performed using GraphPad Prism version 4.00 for Windows, GraphPad Software (San Diego, CA). Multiple groups were compared using one-way analysis of variance followed by a Tukey's multiple comparison test. Two groups were compared using a two-tailed, unpaired t-test. A p value of ≤ 0.05 was considered to be statistically significant.

## Authors' contributions

JB participated in the design of the study, assisted with the DC-T cell co-cultures, data analyses, and drafting of the manuscript. WC carried out and/or assisted with all of the experiments for this study. TT-K conceived of the study, participated in its design and coordination, assisted in data analyses, performed the statistical analyses, and assisted in drafting of the manuscript. All authors read and approved the final manuscript.
